# Do referrals improve the representation of women in mobile phone surveys?^☆^

**DOI:** 10.1016/j.jdeveco.2023.103077

**Published:** 2023-05

**Authors:** Steven Glazerman, Karen A. Grépin, Valerie Mueller, Michael Rosenbaum, Nicole Wu

**Affiliations:** a Innovations for Poverty Action, United States; b School of Public Health, University of Hong Kong, United States; c School of Politics and Global Studies, Arizona State University and International Food Policy Research Institute, United States

**Keywords:** Gender representation, Mobile phone surveys, Referral, Survey methods

## Abstract

Random digit dial surveys with mobile phones risk under-representation of women. To address this, we compare the characteristics of women recruited directly with those of women recruited through referrals from male household members. The referral process improves representation of vulnerable groups, such as young women, the asset poor, and those living in areas with low connectivity. Among mobile phone users, we show a referral (rather than a direct dial) protocol includes more nationally representative proportions of women with these attributes. While seeking intra-household referrals may improve representation, we show that it does so at a higher cost.

## Highlights

• Passing the phone in RDD surveys improves representation of women.
• Referrals reach more youth, the asset poor, and those living in low connectivity.
• Costs affect scalability of referrals given low completion rates.

## 1 Introduction

The COVID-19 pandemic forced many researchers working in low- and middle-income countries (LMICs) to switch to collecting data through mobile phone surveys amid social distancing and travel restrictions. Before the pandemic, Random Digit Dial (RDD) surveys were widely used for public opinion surveys (Gallup, 2008) but they were uncommon in economics and public health (Feng et al., 2018), where using data sourced from representative household surveys conducted in-person is considered the gold standard. In RDD surveys, respondents are sampled through dialing randomly generated phone numbers. More recently, the practice has evolved to first purchasing lists of active phone numbers of mobile phone subscribers and then randomly calling the numbers on the list to construct the sample.

RDD surveys provide a means to recruit samples representative of the mobile phone-owning population in a country with little preparation compared to other nationally representative sampling. The mobile phone modality allows these samples to be surveyed quickly and comparatively cheaply compared to traditional face-to-face modes in LMICs (Dabalen et al., 2016). This is especially relevant during crises, such as the COVID-19, pandemic where face-to-face surveys are unsafe for participants and enumerator alike (Feng et al., 2018).

One of the potential limitations of this approach is the inability to attain truly representative samples since it is only possible to reach respondents who own or use mobile phones (Hu et al., 2010). In LMICs, cellphone ownership is far from universal and is often concentrated among the educated and the wealthy (Silver and Johnson, 2018) and among urban residents (LeFevre et al., 2020). In addition, cell phone ownership does not necessarily result in responses to survey attempts. Response rates in RDD surveys can be low relative to other modes of respondent recruitment (Dillon et al., 2021). Importantly, RDD surveys may also systematically under-sample women (Silver and Johnson, 2018), especially in households that are poor or who follow traditional gender roles where men are expected to own and carry the phone rather than women (Alvi et al., 2020; Hersh et al., 2021). Important gender gaps, with men being more likely to own a phone than women, have been observed in many LMICs (LeFevre et al., 2020). Understanding sources of bias inherent in RDD surveys in LMICs as well as identifying strategies to remove it could improve the effectiveness and efficiency of data collection and the targeting of aid or other social support.

This study tests a method of recruiting survey respondents that could improve the representation of women in RDD surveys: we asked men who were directly dialed to refer us to an eligible woman living in their household by passing the phone to the woman to complete the survey instead. In late 2020, we initiated a longitudinal study in Kenya with the intent of analyzing the gendered impacts of the COVID-19 pandemic. Although the likelihood of including a woman in the sampling frame was only 7 percentage points lower than a man due to phone ownership (Carboni et al., 2021), women in less literate, poorer, and less connected (e.g. rural) households are less likely to own a phone in many LMICs (LeFevre et al., 2020). Thus, we were concerned that the types of women sampled using a standard RDD sampling strategy might not be representative, painting a biased picture of the gendered impacts of the pandemic.

While our referral sampling strategy may increase the number of women who complete the survey, it is unclear whether it allows us to survey a more representative sample of women and the strategy may take more time and be more costly. The question of how to resolve these tradeoffs is thus an empirical one. Therefore, we examine whether the sample characteristics of women recruited through male referrals differed from the women we reached directly through a standard RDD sampling approach. In addition, we measure the additional resources required to reach these women using the referral strategy and the effects of this strategy on the precision of our estimates.

We find that the referral strategy improves our ability to reach women from vulnerable groups, such as young women, poorer women, and those living in areas with low connectivity. Specifically, referrals increase the likelihood of obtaining surveys from women 18–25 years old (8 percentage points), from women living in households without durable walls (6 percentage points) or smartphones (7 percentage points), and from women living in rural areas (7 percentage points). However, while referrals increased our ability to reach more women, the approach requires more time on the phone and more attempts per completed survey, and therefore costs almost twice as much to return an equivalent sample size.

Although the visibility of women in specific subpopulations improves under the referral strategy, whether RDD surveys can be representative at the national level under certain research designs remains an open question (Stuart and Anna, 2017; Stuart et al., 2018). The literature focusing on “hard-to-reach” populations, such as drug users, sexual minorities, and undocumented migrants, typically use techniques like snowball sampling or respondent-driven sampling which are based on referrals inside a network starting with nonrandomly chosen nodes (Goodman, 1961; Heckathorn and Douglas, 2002). In our context, the selection of eligible members stems from a household that was dialed randomly. Much of the uncertainty hinges on the universality of cellphone ownership and access. When we restrict the sample in the national survey to mobile-phone owners, the referral protocol (versus the standard direct-dial approach) approached nationally representative proportions of women in households living in most regions of Kenya. Yet, we still observe that the proportion of women who attended secondary school and who were employed at the time of the interview was greater by 23 percentage points in the referral sample than the proportion of women with those characteristics in the national survey. While within-household referrals may improve the degree to which RDD surveys produce nationally representative samples of women, it may not entirely address the gap in representativeness between an RDD and an equivalent face-to-face probability sample with high response rates. However, in contexts where face-to-face surveying is challenging or not feasible, such as future public health emergencies or humanitarian settings, utilizing referrals could be considered to improve the representation of women.

## 2 Research design

**Fig. 1:**
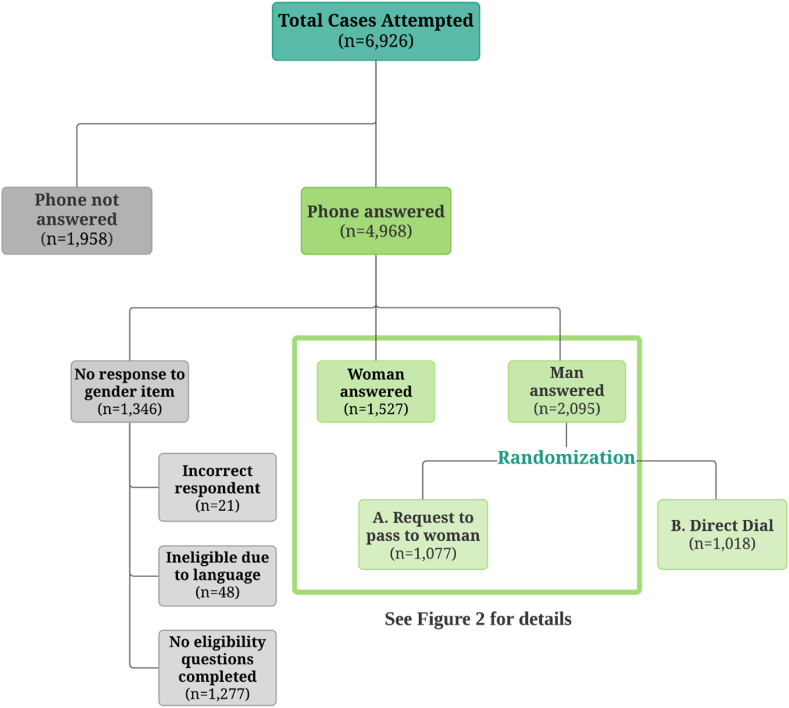
Flow Diagram of Total Cases Attempted and Answered *Note***:** After the call is answered, we first identify if the person who picked up the phone is the owner of the phone number (correct respondents). Twenty-one respondents indicated they are not the owners and that we would not able to reach the owners during the data collection. Forty-eight respondents were not able to complete the survey using either English or Kiswahili. Among the 1277 cases where no eligibility questions were completed, 666 refused to continue the survey at the initial introduction question for different reasons, and 611 ended the call before finishing the survey's eligibility screening questions (including 24 cases that refused to answer their gender).

This study was embedded into an RDD survey that was fielded in Kenya in November to December 2020 that recruited participants into the baseline round of a longitudinal study of gendered response to COVID.^1^ The main objective of the longitudinal survey was to evaluate the gendered health, social, and economic impacts of the pandemic on households (Mueller et al., 2021).^2^ For this analysis, our focus is on the recruitment of women into the sample. Among respondents who first answered the call from the enumerator and identified themselves as male, we randomly selected half of them to be in this referral study (Fig. 1). The other half were interviewed for other purposes but were not included in this analysis.

**Fig. 2:**
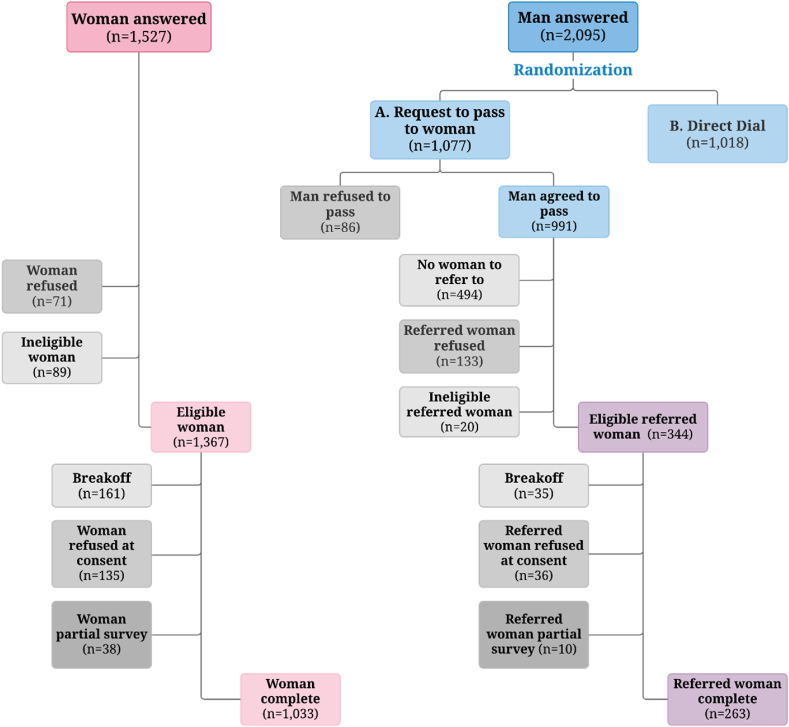
Flow Diagram of Female Respondents in Referral Experiment *Note***:** Breakoff indicates that an eligible respondent did not refuse but exited the survey without starting the survey, for reasons such as respondents rescheduled the interview after consenting to participate but not following through on the interview. “Refused at consent” includes respondents who refuse to accept the audio recording of consent which is required by law for surveys conducted in Kenya.

Because the phone numbers used in the original RDD sample were identified through random selection, we can directly compare women who were directly dialed to those who were referred by a male household member and attribute the differences to the experimental sampling protocol itself (direct dial vs. referral, hereafter Pass the Phone). It should be noted both protocols, Direct Dial and Pass the Phone, result in analytic samples that are subsets of those targeted by each protocol, because of nonresponse, refusal to pass the phone, refusal to accept the referral, or the fact that a woman simply might not be present at the time of the phone call. Fig. 1, Fig. 2 show how many women entered the analytic sample, which women did not, and why. Table A1 details these response rates using AAPOR standard definitions.

**Table 1:**
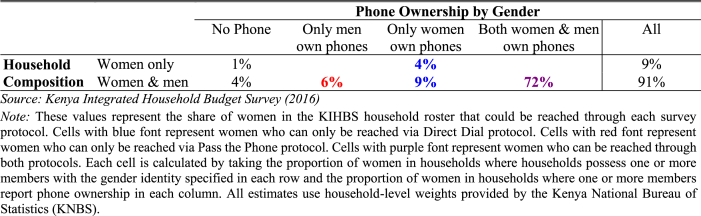
Distribution of women in Kenya: Household composition and phone ownership by gender.

In addition to comparing women recruited through the two protocols for this survey, we compare both groups to a sample of women from a nationally representative household survey conducted in Kenya using face-to-face recruitment before the pandemic. The survey is described in more detail below. Comparisons of the women reached through the Direct Dial versus Pass the Phone inform whether one recruitment protocol is different from the other in terms of the demographic composition of women recruited into the sample. By further comparing women from the RDD survey to the nationally representative household survey, we can determine whether one protocol is better than the other in terms of reducing bias relative to a nationally representative benchmark.

## 3 Data and methods

### 3.1 RDD survey

In the RDD survey (November–December 2021), we recruited women using two separate recruitment strategies: Direct Dial (1033 women) or Pass the Phone (263 women). Each woman who consented to complete the survey was asked a series of questions about household demographics, individual awareness of COVID-19 risk, own mental health, schooling outcomes of child household members, household access to health services, individual time use and employment, household food security, perceptions of social norms, security, individual and joint decision-making, asset ownership, social support since the pandemic, and knowledge of COVID-19 policy responses.

As with any RDD study, not all respondents that were called were included in the final sample due to factors such as non-response or ineligibility. The contact rate across all attempts was 72 percent (Fig. 1), which is relatively high in comparison to other RDD surveys in LMICs (Greenleaf et al., 2020; Dillon et al., 2021). For example, this ranks second to Burkina Faso (75 percent), with other East African countries in a recent multi-country RDD survey conducted during the COVID-19 pandemic, such as Rwanda, Uganda, and Zambia having contact rates of 51, 62, and 64 percent, respectively (Dillon et al., 2021).

There are additional steps between the phone being answered and the questionnaire being administered, where potential respondents may drop out of the final sample (Fig. 2). In the recruitment process, 86 men who were directly dialed refused to pass the phone to a woman in the household, while 991 (92 percent of those asked) agreed to do so. Of the men who agreed to pass the phone, about half, after callbacks were completed, still said that there was not a woman in the household available to come to the phone^3^ and another 13 percent where the referred women subsequently refused to accept the phone that was passed to them.

Before the questionnaire was administered to the women, women could be determined to be ineligible,^4^ refuse to continue with the survey, or breakoff from the interview (e.g., the respondent exited the survey before completing it by rescheduling the interview after the consent script or if the phone connection dropped for other reasons). There were no statistically significant differences in refusal rates between eligible women facing the different recruiting protocols (not shown here). Similarly, there were no statistically significant differences in completing the survey conditional on reaching a woman via the Pass the Phone.

Since the objective of the analysis is to examine the extent to which the demographic, wealth, and location characteristics differ between the women who completed interviews through the two protocols, we created the following variables: age distribution of women (binary categorical variables for each of the following age categories: 18–25, 26–35, 36–45, 46–55, or over 55 years old), marital status (binary, married or not), educational attainment (binary, attended secondary school or not), employment status (binary, had paid employment in the past seven days or not), and the respondent's relationship to the head of the household (binary, was head of household or not). Next, binary outcome variables were constructed from the survey data to describe the wealth and composition of the household, indicating whether the woman resided in a structure with a durable floor or wall or not, whether her household had access to a smartphone and electricity. In addition, outcome variables for household size and the number of young children (0–5 years old), primary-school-aged children (6–13 years old),^5^ and (18 years old and above) adults in the household were constructed. Finally, eleven binary outcome variables were generated to evaluate differences in the location of the interview across recruitment protocols: whether the woman was surveyed alone, whether the woman was surveyed at home, whether the woman lives in an urban location, and one binary variables for each of the regions of residence (Central, Coast, Eastern, Nairobi, North Eastern, Nyanza, Rift Valley, and Western).

### 3.2 Kenya household survey

We use the 2016 Kenya Integrated Household Budget Survey (KIHBS) collected by the Kenya National Bureau of Statistics (KNBS) to serve as a benchmark against which to measure how well the RDD samples (Direct Dial or Pass the Phone) compare to a nationally representative sample. In the KIHBS, approximately, 21,733 households were surveyed, containing 23,869 adult women. The survey collected a wealth of information about the household from their annual consumption expenditures to indicators of nutrition and health. To compare the demographic characteristics across samples, we construct the same variables as detailed above at the individual and household level.^6^

One advantageous feature of the KIHBS is the ability to map households according to gender composition of the adults and to determine whether individual men or women in the household own their own mobile phones – two important characteristics for understanding the representation problems that this protocol study is meant to address. Table 1 suggests that most Kenyan women (72 percent) can be found in households where both men and women own their own mobile phones. Only 5 percent of women are in households where neither a male nor a female adult own a phone, which is encouraging as it suggests that RDD surveys can theoretically reach households with 95 percent of Kenyan women. However, 6 percent of Kenyan women in these households can only be reached by referral. Another 13 percent of Kenyan women can only be reached by direct dial because they live in a household where there is no phone owning male.

The KIHBS data also allow us to determine whether women in the sampling frames of the referral and direct dial protocols have different observable characteristics. Beyond just estimating the size of the population that can be reached in each arm, the KIHBS data allows us to estimate the expected differences between direct dial and referral sampling. Table 2 shows these differences. It compares women who could be reached by Direct Dial in column [1] to women who can only be reached by Pass the Phone in column [2] to women who cannot be reached by either protocol [3]. The differences between [1] and [2] show that women who are sampled by the referral protocol are expected to be meaningfully different on almost every observable demographic characteristic: younger, less likely to be married, less educated, less likely to be employed, less likely to be the household head, more likely to live in a larger household, less asset rich, and more likely to be in Nairobi. However, both samples differ on similar dimensions to the group of women who cannot be contacted through either the direct-dial or referral protocols. The difference between [1] and [3] suggests that an RDD sample would have meaningful and statistically significant differences to accurately represent the Kenyan population. It would have meaningfully different coverage of a segment of the country that is older, married, more educated, employed, and in greater possession of asset wealth.

**Table 2:** Female characteristics by KIHBS sampling frame.

	[1] Direct-Dial	[2] Referral Only	[3] No Phone	[1] - [2] Difference: Sampling Approaches	[1] - [3] Difference: Coverage
Age, 18-25	0.24	0.32	0.36	0.08***	0.12***
				(0.01)	(0.01)
Age, 26-35	0.30	0.29	0.14	-0.01	-0.16***
				(0.01)	(0.01)
Age, 36-45	0.21	0.14	0.11	-0.07***	-0.11***
				(0.01)	(0.01)
Age, 46-55	0.12	0.10	0.08	-0.03***	-0.04***
				(0.01)	(0.01)
Age, 56+	0.13	0.15	0.31	0.02***	0.19***
				(0.01)	(0.01)
Married	0.64	0.82	0.38	0.18***	-0.26***
				(0.01)	(0.01)
Secondary education	0.43	0.13	0.20	-0.29***	-0.23***
				(0.01)	(0.01)
Employed	0.41	0.22	0.19	-0.19***	-0.23***
				(0.01)	(0.01)
Household head	0.32	0.13	0.37	-0.20***	0.04***
				(0.01)	(0.01)
Spouse of household head	0.48	0.74	0.23	0.26***	-0.25***
				(0.01)	(0.01)
Other relationship to household head	0.19	0.13	0.40	-0.06***	0.21***
				(0.01)	(0.01)
Household, number of young children	0.77	1.17	0.86	0.40***	0.09***
	(0.90)	(1.01)		(0.02)	(0.02)
Household, number of primary school aged children	1.15	1.38	1.28	0.24***	0.13***
	(1.20)	(1.29)		(0.03)	(0.02)
Household, number of adults	2.58	2.68	2.74	0.10***	0.16***
	(1.38)	(1.58)		(0.02)	(0.03)
Household size	5.03	5.72	5.46	0.70***	0.44***
	(2.54)	(2.96)		(0.05)	(0.05)
Asset, durable floor	0.55	0.18	0.22	-0.38***	-0.33***
				(0.01)	(0.01)
Asset, durable wall	0.55	0.21	0.26	-0.34***	-0.29***
				(0.01)	(0.01)
Asset, electricity	0.42	0.11	0.13	-0.31***	-0.29***
				(0.01)	(0.01)
Urban	0.43	0.24	0.22	-0.19***	-0.21***
				(0.01)	(0.01)
Region, Central	0.13	0.04	0.05	-0.08***	-0.07***
				(0.01)	(0.00)
Region, Coast	0.12	0.14	0.12	0.03***	0.00
				(0.01)	(0.01)
Region, Eastern	0.18	0.13	0.17	-0.05***	0.00
				(0.01)	(0.01)
Region, Nairobi	0.03	0.01	0.00	-0.02***	-0.03***
				(0.00)	(0.00)
Region, North Eastern	0.05	0.10	0.08	0.05***	0.03***
				(0.01)	(0.00)
Region, Nyanza	0.14	0.13	0.12	-0.02**	-0.02***
				(0.01)	(0.01)
Region, Rift Valley	0.27	0.34	0.35	0.07***	0.08***
				(0.01)	(0.01)
Region, Western	0.09	0.12	0.10	0.03***	0.01*
				(0.01)	(0.01)
Joint test				0.00***	0.00***
N	16537	2728	4562	19265	21099

Note: Outcomes not included here are indicators for whether the respondent is missing the five age categorical variables, the three relationship to the household head variables, and indicators for the household having a durable floor or wall. All of these missing indicators have rare occurrences, i.e. were less than or equal to 1 percent. The joint equivalence row reports the p-value of the F-test of joint significance of all explanatory variables including the indicators for missingness. Employed indicates the respondent reported working one or more hours in the past seven days. Secondary education represents the proportion of respondents who attended secondary school. Standard deviations in parentheses. Robust standard errors in parentheses for the difference column. *** p<0.01, ** p<0.05, * p<0.10

These data from the KIHBS on household composition and phone ownership allow us to differentiate between two types of misrepresentation of women in statistical inference from RDD samples: selection bias and coverage bias. Selection bias arises when women who select not to complete the survey have different outcomes than the rest of the women in the target population. In our context this includes women who refuse to answer the phone, complete the survey, or whose male household members refuse to pass the phone. Coverage bias arises when women who cannot be contacted have different outcomes than women from connected households. In our context this includes certain types of households that lack working mobile phones.

To estimate the magnitudes of these biases, we first compare the characteristics of women from the RDD samples to the entire KIHBS sample of women. Differences between these two samples can arise from variation in the characteristics associated with mobile phone ownership (which leads to coverage bias) as well as respondent willingness to participate in surveys (which leads to selection bias). We, therefore, next restrict comparisons of the RDD samples to subsamples of women from the KIHBS based on phone ownership to uncover sources of selection bias. For example, we can compare women in the Direct Dial sample to the KIHBS sample of women who own phones to determine whether the protocol produces a sample that is at least representative for those it can logically reach. For women in the Pass the Phone sample, we can compare with the KIHBS subsample of households where women live with a man who owns a mobile phone.

We should note a few key limitations in using the KIHBS survey as the benchmark against which to compare the RDD samples. The first discrepancy pertains to mode effects, in terms of how the questions may have been answered across the different surveys. The second relates to the four-year lag in comparisons made between the RDD and national surveys. This means that differences observed between the samples could be the result of sample selection and coverage effects noted above, or they could be due to changes that occurred due to the interview modality or due to changes that occurred over time in the composition of households in Kenya.

### 3.3 Empirical approach

We propose two hypothesis tests regarding the representation of women in RDD samples. The first hypothesis is that the choice of referral protocols influences the sample composition. To test this hypothesis, we directly compare the mean characteristics of Direct Dial with Pass the Phone samples. Statistical significance is assessed using: i) t-statistics based on the difference in means across the two samples, and ii) F-statistics from tests of joint equivalence. The tests of joint equivalence involve estimating the following ordinary least squares regression for a sample that contains data from surveys completed via both Direct Dial and Pass the Phone samples:

(1) $${Pass\;the\;Phone}_{i}=\propto +\beta X_{i}+\varepsilon_{i}$$

where *Pass the Phone* is a binary dependent variable for whether the respondent was recruited through male respondent referrals; and *X* is a vector of the characteristics discussed above, as well as indicators for whether the respondent is missing the five age categorical variables, the three relationships to the household variables, and the durable floor or wall variables.^7^ The joint equivalence test is then estimated after running the regression above, using an F-statistic formed from the test of whether each coefficient in the regression is jointly equal to zero. We report the F-statistic from the joint equivalence test in all the tables.

The second hypothesis is that the choice of recruitment protocols produces a sample that is more representative of the population at the national level. To evaluate this, the same tests as above are performed except the analytic sample includes both the RDD data and the KIHBS data. We compare the characteristics of women from the KIHBS to the characteristics of women in the Pass the Phone and Direct Dial samples. We further evaluate the differences across samples using all women from KIHBS and then repeat the analysis restricting the sample to the appropriate comparison samples. If our second hypothesis holds, we would expect to see fewer statistical differences in the variable means (individually and jointly) in the samples that are more nationally representative. It should be noted that the realized sample sizes for the two protocols are not the same. Therefore, there is a smaller minimum detectable effect between the reference sample and the Direct Dial sample than a corresponding difference between the reference sample and the Pass the Phone sample. For that reason, we focus on magnitudes of difference rather than the number of statistically significant differences. Appendix Table A2 shows the realized minimum detectable effects for estimates of bias (difference between reference sample and protocol sample) for each protocol. These values are higher for the smaller, Pass the Phone protocol, but still within reasonable levels in terms of economic importance. They do not suggest meaningful differences in interpretation if the Pass the Phone protocol had the same sample size as the Direct Dial sample size.

## 4 Results

### 4.1 Differences by recruitment protocol

The Pass the Phone recruitment protocol produced a different sample of women than those recruited through the Direct Dial protocol (Table 3), supporting our first hypothesis. Some of these differences are related to household characteristics, following logically from the method of contact. Women in the referral sample were more often younger (42 versus 34 percent were 18–25 years old), married (59 versus 49 percent), and the spouse of the household head (56 versus 46 percent) rather than themselves being the household head (16 versus 27 percent).^8^ These differences, all statistically significant, are partly explained due to a referral requiring a male household member to be present. Marriage is a common reason for cohabitation of men and women. By reaching more women in mixed-gender households, the referral protocol would naturally produce more male-headed households, which means fewer sample members who are themselves household heads. Consistent with this, Direct Dial is the only way to reach women-only, and hence women-headed, households.

**Table 3:** Female respondent characteristics by recruitment protocol.

	[1] Pass the Phone	[2] Direct-Dial	[2] – [1] Difference
**Age, categorical indicators**			
Age, 18-25	0.42	0.34	−0.08**
			(0.03)
Age, 26-35	0.35	0.38	0.03
			(0.03)
Age, 36-45	0.16	0.16	0.01
			(0.03)
Age, 46-55	0.05	0.08	0.04**
			(0.02)
Age, 56+	0.03	0.04	0.01
			(0.01)
Married	0.59	0.49	−0.10***
			(0.03)
Secondary education	0.80	0.83	0.03
			(0.03)
Employed	0.59	0.58	−0.01
			(0.03)
Urban	0.44	0.50	0.07*
			(0.03)
**Relationship to household head**			
Household head	0.16	0.27	0.11***
			(0.03)
Spouse of household head	0.56	0.46	−0.09***
			(0.03)
Other relationship to household head	0.29	0.27	−0.02
			(0.03)
**Asset measures**			
Durable floor	0.57	0.58	0.01
			(0.03)
Durable wall	0.65	0.71	0.06*
			(0.03)
Smart phone	0.79	0.86	0.07***
			(0.03)
Electricity	0.84	0.87	0.03
			(0.03)
Household composition			
Household size	4.77	4.71	−0.06
			(0.16)
Household, number of young children	0.93	0.77	−0.17**
			(0.07)
Household, number of primary school-aged children	0.90	0.94	0.04
			(0.08)
Household, number of adults	2.56	2.58	0.03
			(0.08)
Surveyed, alone	0.34	0.43	0.09***
			(0.03)
Surveyed, at home	0.60	0.65	0.05
			(0.03)
**Region**			
Central	0.15	0.16	0.01
			(0.03)
Coast	0.10	0.08	−0.02
			(0.02)
Eastern	0.11	0.12	0.01
			(0.02)
Nairobi	0.16	0.24	0.09***
			(0.03)
North Eastern	0.02	0.01	−0.01
			(0.01)
Nyanza	0.14	0.11	−0.03
			(0.02)
Rift Valley	0.26	0.19	−0.07**
			(0.03)
Western	0.07	0.08	0.01
			(0.02)
Joint equivalence			0.00***
N	263	1033	1296

*Note:* Variables omitted from the table are indicators for whether the respondent is missing the five age categorical variables, the three variables for relationship to the household head, and indicators for the household having a durable floor or wall. All of these missing indicators have rare occurrences, i.e. were less than or equal to 1 percent. The joint equivalence row reports the p-value of the F-test of joint significance of all explanatory variables including the indicators for missingness. Employed indicates the respondent reported working one or more hours in the past seven days. Secondary education represents the proportion of respondents who attended secondary school. Robust standard errors in parentheses for the difference column.

***p < 0.01, **p < 0.05, *p < 0.10.

Region and asset ownership tell a different story. Table 3 suggests that the Pass the Phone protocol may allow researchers to improve the targeting of women from income-vulnerable households, in terms of those with lower asset wealth, a greater number of dependents, and living in rural communities. Women recruited through the Pass the Phone protocol were less likely to live in housing with a durable wall (65 versus 71 percent, significantly different at the 10 percent level) and less likely to have access to a smartphone (79 versus 86 percent). They had a greater number of young children (0.93 versus 0.77). Geographic representation was also different, as women in the Pass the Phone sample were 7 percentage points less likely to live in an urban area, 9 percentage points less likely to live in Nairobi, and were 7 percentage points more likely to live in the Rift Valley. The joint equivalence tests confirm that the composition of the female respondents under both protocols statistically differ at the 1 percent critical level.

### 4.2 Differences between RDD and national sample

Both protocols of the RDD survey produced samples of women that differ from the national survey. Table 4 compares women in the RDD Direct Dial sample to women surveyed in the KIHBS. Table 5 does the same for the RDD Pass the Phone sample. All joint equivalence tests indicate that the composition of the RDD and national household survey samples statistically differ.

**Table 4:** Comparisons of female respondent characteristics in direct dial protocol to respondents on national survey.

	[1]	[2]	[3]	[1] – [2]	[1] – [3]
	Direct-Dial	KIHBS	KIHBS phone-owning women	Difference: All households	Difference: Households with comparable phone ownership
**Age, categorical indicators**					
Age, 18-25	0.34	0.28	0.26	0.06***	0.08***
				(0.02)	(0.02)
Age, 26-35	0.38	0.28	0.30	0.10***	0.07***
				(0.02)	(0.02)
Age, 36-45	0.16	0.18	0.20	−0.02	−0.04***
				(0.01)	(0.01)
Age, 46-55	0.08	0.11	0.12	−0.03***	−0.04***
				(0.01)	(0.01)
Age, 56+	0.04	0.15	0.12	−0.11***	−0.08***
				(0.01)	(0.01)
Married	0.49	0.60	0.62	−0.11***	−0.14***
				(0.02)	(0.02)
Secondary education	0.83	0.42	0.49	0.41***	0.34***
				(0.01)	(0.01)
Employed	0.58	0.39	0.45	0.19***	0.13***
				(0.02)	(0.02)
**Relationship to the household head**					
Household head	0.27	0.30	0.32	−0.04**	−0.05***
				(0.01)	(0.01)
Spouse of household head	0.46	0.47	0.49	−0.01	−0.03
				(0.02)	(0.02)
Other relationship to household head	0.27	0.22	0.19	0.05***	0.08***
				(0.01)	(0.01)
**Household composition**					
Household size, total members	4.71	4.92	4.72	−0.21***	−0.02
				(0.08)	(0.08)
Household, number of young children	0.77	0.78	0.73	−0.02	0.04
				(0.03)	(0.03)
Household, number of primary school-aged children	0.94	1.08	1.02	−0.14***	−0.08**
				(0.04)	(0.04)
Household, number of adults	2.58	2.57	2.51	0.02	0.07*
				(0.04)	(0.04)
**Asset measures**					
Durable floor	0.58	0.52	0.63	0.06***	−0.04***
				(0.02)	(0.02)
Durable wall	0.71	0.51	0.60	0.20***	0.11***
				(0.01)	(0.02)
Electricity	0.87	0.42	0.51	0.45***	0.36***
				(0.01)	(0.01)
Urban	0.50	0.39	0.46	0.11***	0.04***
				(0.02)	(0.02)
**Region**					
Central	0.16	0.13	0.15	0.03***	0.01
				(0.01)	(0.01)
Coast	0.08	0.09	0.09	−0.01	−0.01
				(0.01)	(0.01)
Eastern	0.12	0.14	0.14	−0.02**	−0.02**
				(0.01)	(0.01)
Nairobi	0.24	0.11	0.14	0.13***	0.10***
				(0.01)	(0.01)
North Eastern	0.01	0.03	0.02	−0.01***	−0.01*
				(0.0)	(0.0)
Nyanza	0.11	0.13	0.13	−0.02**	−0.02*
				(0.01)	(0.01)
Rift Valley	0.19	0.26	0.24	−0.07***	−0.05***
				(0.01)	(0.01)
Western	0.08	0.10	0.09	−0.02**	−0.01
				(0.01)	(0.01)
Joint test				0.00***	0.00***
N	1033	23,869	16,537	24,902	17,570

*Note:* Outcomes not included here are indicators for whether the respondent is missing indicators for the household having a durable floor or wall. All of these missing indicators have rare occurrences, i.e. were less than or equal to 1 percent. The joint equivalence row reports the p-value of F-test of joint significance of all explanatory variables. Robust standard errors in parentheses for the difference column. ***p < 0.01, **p < 0.05, *p < 0.10.

**Table 5:** Comparisons of female respondent characteristics in Pass the phone protocol to respondents on national survey.

	[1]	[2]	[3]	[1] - [2]	[1] - [3]
	Pass the Phone	KIHBS	KIHBS women in households with phone-owning men	Difference: All households	Difference: Households with comparable phone ownership
**Age, categorical indicators**					
Age, 18-25	0.42	0.28	0.31	0.14***	0.11***
				(0.03)	(0.03)
Age, 26-35	0.35	0.28	0.29	0.07**	0.06*
				(0.03)	(0.03)
Age, 36-45	0.16	0.18	0.18	−0.02	−0.02
				(0.02)	(0.02)
Age, 46-55	0.05	0.11	0.11	−0.06***	−0.06***
				(0.01)	(0.01)
Age, 56+	0.03	0.15	0.11	−0.12***	−0.08***
				(0.01)	(0.01)
Married	0.59	0.60	0.73	−0.01	−0.13***
				(0.03)	(0.03)
Secondary education	0.80	0.42	0.45	0.38***	0.34***
				(0.03)	(0.03)
Employed	0.59	0.39	0.36	0.20***	0.23***
				(0.03)	(0.03)
**Relationship to the household head**					
Household head	0.16	0.30	0.10	−0.15***	0.06***
				(0.02)	(0.02)
Spouse of household head	0.56	0.47	0.67	0.08***	−0.11***
				(0.03)	(0.03)
Other relationship to household head	0.29	0.22	0.23	0.06**	0.06*
				(0.03)	(0.03)
**Household composition**					
Household size, total members	4.77	4.92	5.50	−0.15	−0.73***
				(0.14)	(0.15)
Household, number of young children	0.93	0.78	0.86	0.15**	0.08
				(0.06)	(0.06)
Household, number of primary school-aged children	0.90	1.08	1.12	−0.18***	−0.22***
				(0.07)	(0.07)
Household, number of adults	2.56	2.57	3.01	−0.01	−0.45***
				(0.07)	(0.07)
**Asset measures**					
Durable floor	0.57	0.52	0.57	0.05	0.00
				(0.03)	(0.03)
Durable wall	0.65	0.51	0.54	0.14***	0.11***
				(0.03)	(0.03)
Electricity	0.84	0.42	0.46	0.42***	0.37***
				(0.02)	(0.02)
Urban	0.44	0.39	0.42	0.05	0.02
				(0.03)	(0.03)
**Region**					
Central	0.15	0.13	0.13	0.02	0.02
				(0.02)	(0.02)
Coast	0.10	0.09	0.10	0.01	0.00
				(0.02)	(0.02)
Eastern	0.11	0.14	0.13	−0.04*	−0.03
				(0.02)	(0.02)
Nairobi	0.16	0.11	0.13	0.04*	0.03
				(0.02)	(0.02)
North Eastern	0.02	0.03	0.03	−0.01	−0.01
				(0.01)	(0.01)
Nyanza	0.14	0.13	0.13	0.01	0.02
				(0.02)	(0.02)
Rift Valley	0.26	0.26	0.26	0.00	0.00
				(0.03)	(0.03)
Western	0.07	0.10	0.10	−0.03**	−0.03*
				(0.02)	(0.02)
Joint test				0.00***	0.00***
N	263	23,869	14,780	24,132	15,043

*Note:* Outcomes not included here are indicators for whether each variable was missing. All of these missing indicators have rare occurrences, i.e. were less than or equal to 1 percent. The joint equivalence row reports the p-value of the F-test of joint significance of all explanatory variables. Robust standard errors in parentheses for the difference column. ***p < 0.01, **p < 0.05, *p < 0.10.

**Direct Dial.** The Direct Dial protocol, which is the standard RDD approach, produces a sample of women that is younger, more educated, more likely employed, and more urban than Kenyan women nationally. This is shown in Table 4, comparing columns [1] and [2]. This comparison with women in the full KIHBS sample illustrates the role of both selection and coverage effects on differences between RDD samples relative to face-to-face survey samples. For example, 34 percent of the Direct Dial sample of women are in the youngest age group (18–25 years old) compared to 28 percent nationally, while only 4 percent are in the oldest group (56 and older) compared to 15 percent nationally. Women in the RDD Direct Dial sample are considerably more likely to have a secondary education than the national sample (83 versus 42 percent) and be employed (58 versus 39 percent). The RDD survey produces a sample of women with different household characteristics as well, notably from smaller households (4.7 versus 4.9 members).

To isolate the role of selection effects from coverage effects among mobile-phone-using individuals and households, we compare columns [1] and [3] in Table 4. Column [3] restricts the KIHBS sample to households with mobile phone-owning women, so it should be more directly comparable to the Direct Dial RDD mobile phone survey. This restriction does not change the sample composition meaningfully, so some important differentials noted above between RDD and the national survey (in age, education, and employment) persist. The main difference is that this restriction on the KIHBS sample makes the RDD sample look a bit more representative in terms of asset wealth (floors, walls, and electricity) and urbanicity. This is consistent with the story that mobile phone service coverage effects underrepresent the poorest women whereas selection effects (behavioral response of not answering the phone or not staying on to give consent) underrepresent older and less educated women.

**Pass the Phone.** The motivation for adding a referral protocol to the RDD survey was to reach those women (6 percent of the total nationally, according to Table 1) who do not own phones but live with men who do. Comparing columns [1] and [2] in Table 5 shows that, again, RDD produces a sample of women who are younger, more educated, urban, and more likely to be employed than women nationally in Kenya. However, this Pass the Phone protocol nearly eliminates the marriage gap and slightly reduces the gap in household size, making it statistically insignificant and reduces gaps in urbanicity and region compared to the Direct Dial sample. Asset ownership results still suggest that the RDD sample with Pass the Phone protocol comprises more asset-rich women than the national sample based on two of the three measures (durable walls and electricity).

As with the Direct Dial sample, we imposed a restriction on the KIHBS sample in column [3] that seeks a more “apples-to-apples” comparison of households in order to remove the coverage effects and isolate selection effects. In this case it includes just those households with women who live with phone-owning men. These differences should be smaller, than the column [1] versus [2] differences. They are smaller for age, education, asset wealth, and region but in some other cases they are not. Notably, the restricted KIHBS sample suggests that Pass the Phone sample should have larger households (5.5 members) with more women being married (67 percent) and more school-age children (1.1) and adults (3.0) present, but the Pass the Phone sample has significantly fewer married women and smaller households than this, making it more comparable to the national survey without sample restrictions. This suggests that selection and coverage effects may be acting in opposite directions, offsetting each other for this protocol.

There is reason to expect that selection works differently from Direct Dial because this protocol has the added behavioral component of men's decisions of whether to pass the phone, and women's decisions to complete the survey being mediated by the fact that their spouse or other male household member is involved. Each of these could change the sample composition in important ways.

**Direct Dial versus Pass the Phone.**Table 3, Table 4, Table 5 suggest that the RDD protocol matters for achieving a representative sample. Neither will completely mimic the national sample, nor should they, given caveats about the confounded differences like time lag between the reference survey and mode differences. However, it is clear that the Pass the Phone protocol produces a different sample of women in terms of household characteristics like household size, composition, headship, and marital status, and reduces some of the representation socio-economic and geographic gaps in more subtle ways. While we separated these protocols for the purposes of this paper, future RDD surveys that seek subpopulations such as women or members of a particular age group would most naturally include both direct-dialed and referred sample members in a combined sample.

**Fig. 3:**
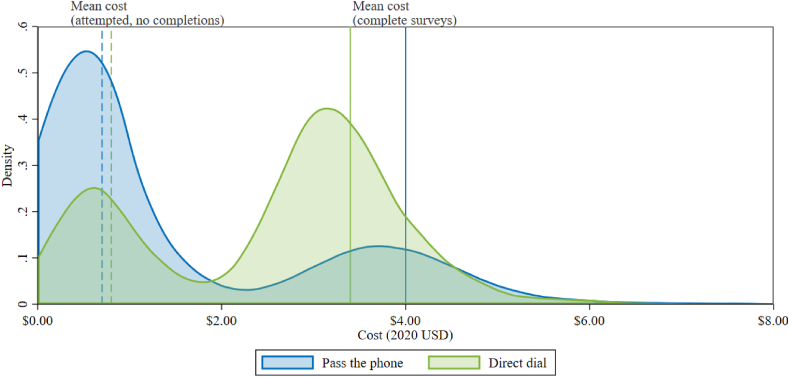
Cost of Interview by Protocol Type *Note*: This figure displays frequency-weighted differences in costs among all cases that reported their gender identity, the sample shown in Fig. 2. Each observation is the total cost related to an individual case (randomly dialed number) over all attempts during the survey duration. The dashed lines report the average costs of the subsample of observations that do not return a complete survey. Most of these cases exited the survey before the consent. The solid lines report the average costs of the sample of observations that return a complete survey. All cases complete the final question of the final module.

**Cost Implications and Sample Efficiency.** Our study finds that a simple referral protocol in which male respondents are asked to refer an eligible woman in their household to complete the survey instead of themselves allowed us to collect data from some women who might have been harder to access otherwise, notably more often married and slightly more urban and less educated women. In addition, we find that the referral strategy allowed us to collect data from a more representative sample than was possible with a standard RDD design. When accounting for enumerator wages, phone credit expenses, and the incentives paid to respondents, obtaining a completed survey through a Direct Dial or Pass the Phone protocol averages $3.78 or $6.20, respectively (Table A3, Fig. 3). The disparity in costs between the two recruitment procedures mainly stems from variation in the likelihood of achieving a completion (0.68 in Direct Dial versus 0.24 in Pass the Phone). Although, neither RDD sampling strategy was able to collect data that was truly representative at the national level, mobile data collections strategies may be advantageous relative to more traditional in-person surveying due to the large cost benefits of RDD surveys and the ability to reach respondents at challenging times such as the COVID-19 pandemic (Gourlay et al., 2021).

One additional factor to consider in these cost calculations is the efficiency loss from sampling and weighting procedures compared to simple random sampling, called the estimated design effect, *DEFF* (Eckman and West, 2016). The design effect measures the loss or gain in precision from the sampling and weighting of more complex designs. Differences in the design effect between each recruitment method allow us to gauge how changes in the sample composition trade off changes in precision of estimates for this approach. As both recruitment protocols use simple random sampling, we compare the component of the estimated design effect that results from unequal weighting (Kish, 1992; Kish, 1992):

(2) $${DEFF}_{w_{r}}=1+cv_{w_{r}}^{2}=1+\frac{\sigma_{w_{r}}^{2}}{\mu_{w_{r}}}$$

${DEFF}_{w_{r}}$ represents the increase in variance due to unequal weights. It is calculated by estimating the relative variance of the weights for each recruitment protocol, where $\sigma_{w_{r}}^{2}$ is the standard deviation of the vector of weights, *w*, in each recruitment protocol, *r*, and $\mu_{w_{r}}$ is the mean weight value for each recruitment protocol. To compute the *weighted* variances of each sample, we use the method adopted by Kastelic et al. (2020). Post-stratification weights are computed using the population counts in each demographic group (gender, age, county). Weights are then top-coded at the 99th percentile. This analysis estimates post-stratification weights separately for each recruitment protocol.

In both cases, the design effect is greater than 1, which implies that both protocols have increased variances and efficiency loss compared to simple random samples without coverage or selection bias (Eckman and West, 2016). With a design effect of 1.02 (Pass the Phone) relative to 1.31 (Direct Dial), accounting for the weights results in increased variance for estimates in the Direct Dial sample compared to the Pass the Phone sample. These factors suggest recruiting additional women through Pass the Phone may improve precision both through increasing the sample size and by reducing variation attributable to the sample weights. To demonstrate the implications of differences in efficiency loss on our cost estimates, we compute the effective sample size, a measure which illustrates what the size of a simple random sample would need to be to produce the same variance. If each protocol returned 1000 respondents, the effective sample size for the Pass the Phone sample would be 980 (1,000/1.02) compared to the effect sample size for the Direct Dial sample of 763 (1000/1.31). Even when considering the gains in precision due the sample size, it would still be more cost effective to increase the effective sample size by 1 using the Direct Dial protocol (1.31 × 3.78 = $4.95) compared to the Pass the Phone protocol (1.02 × 6.20 = $6.32). Increasing the effective sample size is 28 percent (6.32 ÷ 4.95 = 1.28) more expensive in the Pass the Phone protocol compared to increasing the sample size where the Pass the Phone protocol is 64 percent (6.20 ÷ 3.78 = 1.64) more expensive. Our results remain qualitatively unchanged: although the Pass the Phone sample is more expensive, adding women to the sample who are more likely to be younger, more educated, employed, and living in urban areas may justify the increased cost of the Pass the Phone protocol for certain research questions.

## 5 Conclusion

There is growing interest in using RDD mobile phone surveys to collect health and economic data in LMICs, especially in contexts where data collection is difficult. However, there are important concerns about the representativeness of such data. We examined the feasibility of reaching women using RDD surveys in Kenya, where mobile phone access is widespread and gender inequities in ownership are less severe relative to other LMICs.

One motivation for incurring the additional costs required for the Pass the Phone protocol is the desire to improve the representation of women. Understanding the extent women possess autonomy in the household or have influential power in the decision-making process requires interviewing married women. Cellphone ownership and usage among married women can vary with social norms (Alvi et al., 2020; Hersh et al., 2021). We find that we are much more likely to obtain married women in the sample through referral than direct dialing (a 10 percentage point difference), and the proportion of married women in the referral sample converges to the proportion of married women in the national survey. However, the greater capacity to improve the representation of married women comes at substantive monetary cost. Furthermore, there appear to be no statistically significant differences in responses when comparing the decision-making and intrahousehold conflict outcomes among married women in both protocols (Table A4).

Similarly, even though the proportion of married women in the recruitment sample equals the proportion present in the national population, the referred women are not necessarily more nationally representative. When comparing their attributes to women interviewed in a national household survey, women with lower education levels and women who engage primarily in unpaid or domestic work remain underrepresented. Referral does not entirely help solve the coverage bias issue inherent in survey data. RDD approaches also remain prone to selection issues irrespective of the protocol, as the observable attributes of the sample of women acquired through direct dial differ from those of women reported to own phones at the national level.

Policymaker demand for inference about national populations and constraints on in-person sample recruitment, such as cost or public health emergencies, will continue to create interest in RDD methods. Although our findings focus on the apparent biases present in RDD sampling frames among women, the insights should also be useful for researchers seeking better representation of other hard to reach populations that vary *within* a household, such as both very old and very young individuals or widows. But, more research is needed to better understand how to increase representativeness of characteristics that vary across households and can affect mobile phone ownership, such as urbanicity or income. Future research is warranted to discover ways of improving this low-cost data collection mode through the optimal combination of direct and referral protocols, statistical adjustment, and the right mix of messages and incentives and other sample recruitment strategies to increase contact rates and lower refusal rates. This paper advances our knowledge about the mechanisms that produce more or less representative samples of the populations of interest as well as reflects on the precision underlying the estimates obtained under various protocols.

## Credit author statement

All authors equally contributed to the conceptualization, data curation, methodology, formal analysis, methodology, and writing of the manuscript.

## Figures and Tables

**Table A.1:** Disposition Codes and Call Outcome Rates by Recruitment Protocol

				Direct Dial		Pass the Phone	
Panel 1: Prior to Eligibility Screening							
Unknown Eligibility							
4.32	Non-working/Disconnected Number	152	2.19%				
3.10 (UH)	No Answer	1806	26.08%				
3.20 (UH)	Answered, No Screener Completed	1636	23.62%				
Panel 2: After Eligibility Screening							
Not Eligible							
4.70	Ineligible			89	5.83%	514	47.73%
Eligible, Non-interview							
2.10 (R)	Refusal/break-off			296	19.38%	71	6.59%
Interview							
1.20 (P)	Partial			38	2.49%	10	.93%
1.10 (I)	Complete			1033	67.65%	263	24.42%
Panel 3: AAPOR Response Rates							
Response Rate 3∗					20.32%		69.65%
Cooperation Rate 1					76.45%		75.57%
Refusal Rate 2∗					5.49%		19.96%
Contact Rate 2∗					26.58%		92.18%

*Note:* AAPOR standard definitions are used to describe the dispositions of each attempt and to calculate the response rates in this table. Additional details on where in the survey protocol potential respondents exited the survey are shown in Fig. 1, Fig. 2.

∗ e, the estimated proportion of unknown eligibility cases that are eligible is estimated separately for each protocol by multiplying (a) the proportion of dials that result in a male (Pass the Phone) or female (Direct Dial) initial respondent by (b) the eligibility rate for each protocol. Attempts to men where females were reported to not be available were considered to be ineligible.

**Table A.2:** Disposition Codes and Call Outcome Rates by Recruitment Protocol

	Pass the Phone			Direct Dial		
	MDE	Point Estimate		MDE	Point Estimate	
Age, 18–25	0.087	0.107	***	0.044	0.082	***
Age, 26–35	0.084	0.057	*	0.045	0.074	***
Age, 36–45	0.064	−0.021		0.035	−0.043	***
Age, 46–55	0.037	−0.063	***	0.026	−0.035	***
Age, 56+	0.031	−0.080	***	0.019	−0.078	***
Married	0.086	−0.135	***	0.046	−0.136	***
Secondary education	0.071	0.344	***	0.036	0.341	***
Employed	0.087	0.231	***	0.046	0.135	***
Household head	0.064	0.059	***	0.041	−0.050	***
Spouse of household head	0.088	−0.115	***	0.046	−0.027	
Other relationship to household head	0.080	0.055	*	0.041	0.077	***
Household Size	0.408	−0.732	***	0.226	−0.017	
Household, number of young children	0.171	0.075		0.085	0.039	
Household, number of primary school aged children	0.193	−0.220	***	0.103	−0.077	**
Household, number of adults	0.201	−0.452	***	0.119	0.072	*
Asset, durable floor	0.087	0.004		0.045	−0.045	***
Asset, durable wall	0.084	0.106	***	0.043	0.113	***
Asset, electricity	0.066	0.374	***	0.034	0.357	***
Urban	0.088	0.015		0.047	0.045	***
Region, Central	0.063	0.021		0.034	0.012	
Region, Coast	0.052	0.003		0.025	−0.011	
Region, Eastern	0.054	−0.026		0.030	−0.023	**
Region, Nairobi	0.066	0.026		0.042	0.100	***
Region, North Eastern	0.024	−0.010		0.010	−0.007	*
Region, Nyanza	0.061	0.015		0.029	−0.020	*
Region, Rift Valley	0.077	0.001		0.036	−0.046	***
Region, Western	0.044	−0.030	*	0.025	−0.007	
N		15,043			17,570	

*Note:* Minimum Detectable Effects (MDEs) included here are estimated by multiplying the standard error of the “[1]-[3]” column in Table 4, Table 5 by 2.80, such that they describe a 95 percent confidence interval and power of 80 percent (Gelman and Hill, 2006). Outcomes not included here are indicators for whether each variable was missing. All of these missing indicators have rare occurrences, i.e. were less than or equal to 1 percent. Robust standard errors used for difference estimates. ***p < 0.01, **p < 0.05, *p < 0.10.

**Table A.3:** Average Costs Among Female Respondents by Recruitment Protocol

	Unknown gender identity	[1] Pass the phone	[2] Direct dial	[2] - [1]
Number of cases	3304	1077	1527	
Number of completions		263	1033	
Completion rate		.24	.68	
Cases required per completion		4.10	1.48	−2.62
Average duration, incomplete cases (minutes)		11.53	13.59	2.06
Average duration, complete cases (minutes)		50.06	40.74	−9.32
Average cost per incomplete case		$.71	$.83	$.12
Average cost per complete survey		$4.01	$3.38	-$.63
Average cost per case	$.33	$1.51	$2.55	$1.04
Cost per completion		$6.20	$3.78	-$2.43

*Note:* Cost per completion calculates the cost required to produce one completion using each protocol, taking into account the costs of cases that do not return a complete survey. This table excludes 1018 male-answered cases completed by men. We include the following in our calculation of costs: 1) staffing costs, 2) monetary incentives for complete surveys, and 3) expenses for phone time. Staffing costs are determined from IPA financial reports. Phone plan costs are variable due to the structure of the plan, but we assumed 10,000-min packages priced at $120.62 since the majority of costs followed this structure. Finally, all respondents were paid an incentive of $0.91 to complete the survey. Costs are produced by using the total survey duration, top-coded to the 99th percentile.

**Table A.4:** Security, Time Use, and Well-Being Outcomes by Recruitment Protocol

	[1] Pass the phone	[2] Direct dial	[2] - [1]
*Panel 1: All Respondents*			
**Mental Health**			
GAD-2 Combined Score	0.84	0.92	0.08
	(1.37)	(1.30)	(0.09)
PHQ-2 Combined Score	0.98	0.99	0.00
	(1.26)	(1.24)	(0.09)
**Time Use (minutes)**			
Minutes spent in work	277.02	270.77	−6.25
	(235.07)	(239.43)	(16.28)
Minutes spent in domestic activities	207.32	195.58	−11.74
	(138.37)	(127.33)	(9.40)
Minutes spent in shopping	21.27	27.65	6.38***
	(34.43)	(40.80)	(2.47)
Minutes spent in religious activities and leisure	183.59	207.43	23.83**
	(147.99)	(151.90)	(10.27)
Minutes spent in eating and drinking	66.96	68.96	2.00
	(38.14)	(43.68)	(2.71)
Minutes spent in health services	8.38	8.39	0.01
	(47.28)	(48.43)	(3.28)
Minutes spent in unpaid child or adult care	88.46	90.90	2.44
	(114.35)	(127.49)	(8.08)
Minutes spent in traveling	44.94	49.76	4.82
	(68.78)	(75.15)	(4.84)
Minutes spent in sleeping	542.05	520.56	−21.50**
	(141.15)	(139.63)	(9.72)
**Food Security**			
Did not have enough food to eat in the past 7 days	0.59	0.55	−0.04
	(0.49)	(0.50)	(0.03)
Days consuming less expensive food	3.81	3.65	−0.16
	(1.73)	(1.85)	(0.16)
Days eating borrowed food	1.32	1.27	−0.05
	(1.78)	(1.75)	(0.16)
Days reducing number of daily meals	3.56	3.34	−0.22
	(2.38)	(2.47)	(0.22)
Days reducing meal portion size	3.47	3.28	−0.18
	(2.53)	(2.58)	(0.23)
Days reducing eaten quantities	2.40	2.37	−0.03
	(2.63)	(2.71)	(0.24)
Joint equivalence			0.00***
N	263	1033	1296
*Panel 2: Married Respondents Only*			
**Household Conflict**			
Household had no argument in past 6 months	0.26	0.28	0.01
	(0.44)	(0.45)	(0.03)
Household argued once or twice in past 6 months	0.29	0.28	0.00
	(0.45)	(0.45)	(0.03)
Household argued monthly and more in past 6 months	0.45	0.44	−0.01
	(0.50)	(0.50)	(0.04)
Perceived more conflict after COVID-19	0.55	0.56	0.01
	(0.50)	(0.50)	(0.04)
Perceived same level of conflict after COVID-19	0.29	0.30	0.02
	(0.45)	(0.46)	(0.03)
**Household Decision-making**			
Respondent involved in money use decision	0.92	0.92	0.00
	(0.27)	(0.27)	(0.03)
Respondent involved in healthcare decision	0.80	0.81	0.01
	(0.40)	(0.39)	(0.04)
Respondent involved in COVID policy adherence decision	0.87	0.87	0.00
	(0.34)	(0.34)	(0.03)
Joint equivalence			0.244
N	263	1033	1296

*Note:* We create the Generalized Anxiety Disorder (GAD)-2 and Patient Health Questionnaire (PHQ)-2 scores from the responses to the questions in the mental health module following previous work (Löwe et al., 2005; Kastelic et al., 2020; Plummer et al., 2016). The anxiety and depression scores range from 0 to 6. The time use outcomes reflect the number of hours spent in the last 24 h in each activity. The food security outcomes are constructed from questions that are often included in the Demographic Health Surveys. The first outcome is a binary indicator that reflects food insecurity, whether the respondent lives in a household that did not have enough food to eat in the last seven days. The remaining five food-related outcomes report the number of days the respondent's household adopted each coping strategy. The mean values are reported for a restricted sample that obtained a value of one for the first food insecurity indicator. The means of the household conflict and decision-making outcomes are reported for the sample of married women. The conflict outcomes are binary variables that reflect the frequency of arguments in the past 6 months, and whether the perception of the respondent was that there was more or an equivalent number of intrahousehold conflicts than prior to COVID-19. For the decision-making outcomes, binary variables are created to indicate whether the women were involved in how money is used, decisions related to health care, or adherence to COVID preventive measures in the household. The respondent is “involved” in a decision if she answered that she either jointly (with other household members) or solely makes the decisions in each category. ***p < 0.01 **p < 0.05 *p < 0.10.

## Data Availability

Data will be made available on request.

## Footnotes

1 The study protocol was approved by the Institutional Review Board at Simon Frasier University (SFU) with a reliance agreement between SFU and Arizona State University (FWA 00009102; IRB Registration Number: IRB00000128). Separate ethical reviews were conducted by Hong Kong University's Human Research Ethics Committee (EA, 200220) and by Maseno University's Ethics Review Committee in Kenya (MSU/DRPI/MUERC/00906/20).

2 The study is included in the American Economic Association RCT registry, “Referral Protocols for Achieving Gender Representation in Random Digit Dial Phone Surveys” (RCT ID: AEARCTR-0007765).

3 We are unable to measure the extent to which the omission of this sample might have skewed the composition of the sample of women in the Pass the Phone group since the men giving the referrals were also not interviewed. Therefore, it is important to interpret the “Pass the Phone” sample as being informed by sampling and gender norms of the man passing the phone.

4 Eligibility criteria included the respondent's gender, their age (18 years old or above), language, and willingness to participate in follow-up surveys, which required documenting the respondent's contact information and name.

5 We omitted one category of children from this list, which captured other children not in primary school. This technically consisted of secondary-school aged school adolescents (14–17 years old) and other children not going to school. In practice, this means that we may underestimate the true number of primary-school aged children living in the household.

6 Summary statistics from the national household survey dataset are meant to represent the overall population. For this reason, we always apply survey weights to formulate summary statistics in using the KIHBS samples. The survey weights are based on a two-stage stratified sampling frame from the 2009 Kenyan Census that were adjusted for KIHBS survey response rates (KNBS, 2016).

7 Item nonresponse is rare, i.e., less than or equal to 1 percent for each variable, and therefore are not displayed in the tables of results.

8 All differences mentioned in the text are statistically significant at the 0.05 level unless otherwise noted.
